# Hospitalized COVID‐19 patients with diabetes have an increased risk for pneumonia, intensive care unit requirement, intubation, and death: A cross‐sectional cohort study in Mexico in 2020

**DOI:** 10.1002/hsr2.1222

**Published:** 2023-04-18

**Authors:** Alexander A. Huang, Samuel Y. Huang

**Affiliations:** ^1^ Department of Statistics and Data Science Cornell University Ithaca New York USA; ^2^ Northwestern University Feinberg School of Medicine Chicago Illinois USA; ^3^ Virginia Commonwealth University School of Medicine Richmond USA

**Keywords:** COVID‐19, diabetes mellitus, ICU, intubation, logistic regression, Mexico, mortality, pneumonia, resource allocation, retrospective cohort study, risk factors

## Abstract

**Background:**

Diabetes mellitus is a chronic health condition that has been linked with an increased risk of severe illness and mortality from COVID‐19. In Mexico, the impact of diabetes on COVID‐19 outcomes in hospitalized patients has not been fully quantified. Understanding the increased risk posed by diabetes in this patient population can help healthcare providers better allocate resources and improve patient outcomes.

**Objective:**

The objective of this study was to quantify the extent outcomes (pneumonia, intensive care unit [ICU] stay, intubation, and death) are worsened in diabetic patients with COVID‐19.

**Methods:**

Between April 14, 2020 and December 20, 2020 (last accessed), data from the open‐source COVID‐19 database maintained by the Mexican Federal Government were examined. Utilizing hospitalized COVID‐19 patients with complete outcome data, a retrospective cohort study (*N* = 402,388) was carried out. In relation to COVID‐19, both univariate and multivariate logistic regression were used to investigate the effect of diabetes on specific outcomes.

**Results:**

The analysis included 402,388 adults (age >18) with confirmed hospitalized COVID‐19 cases with mean age 46.16 (standard deviation = 15.55), 214,161 (53%) male. The outcomes delineated included pneumonia (*N* = 88,064; 22%), ICU requirement (*N* = 23,670; 6%), intubation (*N* = 23,670; 6%), and death (*N* = 55,356; 14%). After controlling for confounding variables diabetes continued to be an independent risk factor for both pneumonia (odds ratio [OR]: 1.8, confidence interval [CI]: 1.76−1.84, *p* < 0.01), ICU requirement (OR: 1.09, CI: 1.04−1.14, *p* < 0.01), intubation (OR: 1.07, CI: 1.04−1.11, *p* < 0.01), and death (OR: 1.88, CI: 1.84−1.93, *p* < 0.01) in COVID‐19 patients.

**Conclusions:**

According to the study, all outcomes (pneumonia, ICU requirement, intubation, and death) were greater among hospitalized individuals with diabetes and COVID‐19. Additional study is required to acquire a better understanding of how diabetes affects COVID‐19 outcomes and to develop practical mitigation techniques for the risk of severe sickness and complications in this particular patient population.

## 1 INTRODUCTION

In Wuhan, China, in December 2019, the COVID‐19 pandemic originally manifested as an unidentified respiratory condition. From there, it quickly spread over the world, arriving in Mexico in February 2020. On March 11, 2020, the World Health Organization labeled it a global pandemic.^1^ More than 7 million SARS‐CoV‐2 cases have been confirmed as of this writing, and more than 300,000 people have died globally as a result. Over 200,000,000 individuals, or roughly 75% of the world's population, have gotten at least one dose of a vaccination.^2^, ^3^ Latin America continues to a high burden of active COVID‐19 cases.^4^, ^5^, ^6^

Most COVID‐19 infected individuals recover without the need for specialized medical care, and many do not exhibit any symptoms.^7^, ^8^ The severity of the disease is modulated by coexisting medical disorders.^9^, ^10^ Pneumonia or respiratory failure are common complications in COVID‐19 cases and can be deadly.^11^, ^12^, ^13^, ^14^

The chance of developing a severe disease from COVID‐19 can rise with age and certain preexisting medical conditions. Comorbidities like hypertension, obesity, diabetes, and smoking are very common in Mexico, which raises the risk of COVID‐19.^15^, ^16^, ^17^ Smoking has been shown to increase the risk of severe complications and poor outcomes in COVID‐19 patients, as it can damage the respiratory system and weaken the immune system, making it harder for the body to fight off the virus.^18^

Obesity and hypertension are two comorbidities that are frequently present in diabetes patients and can raise the risk of respiratory problems.^19^, ^20^, ^21^ Diabetes is thought to increase the incidence of respiratory illnesses, such as COVID‐19, and can have a detrimental effect on the prognosis of patients with respiratory illnesses because chronic inflammation might impair the immune system.^22^, ^23^, ^24^ Additionally, older people, who's poorer immunity makes them more susceptible to COVID‐19, make up a sizable component of the Mexican population.^25^, ^26^

The relationship between COVID‐19 and diabetes is a complex one, with both conditions impacting each other in multiple ways as found in many populational studies. On one hand, COVID‐19 can lead to elevated glucose levels through changes in the immune and coagulation systems. On the other hand, diabetics are at a higher risk of contracting COVID‐19. The chronic nature of diabetes is associated with chronic inflammation, and the underlying mechanisms behind the link between the two conditions is not well understood, but it is believed that an exaggerated immune response in diabetics may contribute to a more severe course of COVID‐19.^27^, ^28^

Understanding the effects of COVID‐19 in various groups is crucial for implementing efficient mitigation measures and correctly triaging patients during an epidemic.^29^, ^30^ There have yet to be sizable studies done in Mexican patients to look at the association between diabetes and COVID‐19 outcomes. There is urgency, given the fact that it is thought that comorbidities like diabetes may raise the risk of fatal consequences from COVID‐19. Using a large, nationwide retrospective cohort, this study attempts to uncover the significant health consequences (pneumonia, intensive care unit [ICU] requirement, intubation, and death) that individuals with diabetes and COVID‐19 are more at risk for.

## 2 METHODS

In this study, data from the Federal Government of Mexico were examined using a retrospective, cross‐sectional cohort methodology. The Epidemiological Surveillance System for Viral Respiratory Diseases of the Mexican Ministry of Health released and validated the data set, and their ethics committees gave their approval.

### 2.1 Data set and cohort selection

The Mexican Ministry of Health created and distributed the COVID‐19 Mexican Patients Data set, which has been used to analyze the COVID‐19 status, demographics, and outcomes of the Mexican population. The data set is a cross‐sectional, intricate, and multistage collection of information from 475 medical and monitoring facilities for viral respiratory diseases. In the current research, which included all hospitalized COVID positive patients from April 14, 2020 to December 20, 2020. The first 1,048,575 adult patients were analyzed due to constraints from excel and of that 402,388 adult (>18 years of age) patients were included that had complete information on ICU stay, intubation, death, and pneumonia rates. Sex, age, nationality, chronic disease status (hypertension, diabetes, and obesity), smoking status, and pregnancy status are all recorded on everyone. The antigen result and whether an antigen sample was taken are registered data for COVID‐19 status. The public was not given access to any information about the patient's progress during their time in the medical facilities.

### 2.2 Determination of COVID‐19

SARS‐CoV‐2 antigen detection on nasal swabs was used to establish the COVID‐19 status. The Mexican government carried out the test at numerous monitoring and medical facilities, which enabled quick findings. The presence of the SARS‐CoV‐2 antigen validated a positive COVID‐19 status, whereas its absence indicated a negative status.

### 2.3 Independent variables

We examined the outcomes of pneumonia, need for an ICU, need for intubation, and mortality. Age, sex, and other comorbidities (hypertension, obesity, and smoking status) were among the confounding factors considered in the analysis. Diabetes, hypertension, and obesity were defined as if the patient had a diagnosis from a provider of any of these conditions. Native was defined by if the patient identified they were from Mexico.

### 2.4 Statistical analysis

For patients who tested positive for the SARS‐CoV‐2 antigen, descriptive statistics were created that included pertinent demographic information and diabetes status. *χ* ^2^ tests were employed for categorical data while *T*‐tests were utilized to compare continuous variables. Initial analyses of the impact of various factors on COVID‐19 results employed univariable models (pneumonia, intubation, ICU requirement, and death). The impact of diabetes on COVID‐19 outcomes was then evaluated using multivariable models that controlled for age, sex, and other comorbidities. After determination of linearity or predictors, independence of observations to avoid bias, and determination of normality of the residuals a logistic regression model would be selected for analysis of the binary outcome variables. Analysis was done via R Statistical Software.

## 3 RESULTS

Table 1 describes the demographic variables and important covariates of COVID‐19 positive patients in the Mexican Patients Data set released by the Mexican Ministry of Health. A total of 402,388 adults (age >18) hospitalized with COVID‐19 were included in the study. In this group 214,161 (53%) were male and 188,227 (47%) were female. The mean age was 46.16 (standard deviation = 15.55). Common comorbidities in this group included 81,604 (20%) patients with hypertension, 66,082 (16%) with diabetes, and 77,003 (19%) with obesity. Twenty‐nine thousand nine hundred ninety‐six (7%) of the individuals were smokers. Eighty‐eight thousand sixty‐four (22%) of the individuals had pneumonia, 10,285 (3%) required ICU level of stay, 23,670 (6%) required intubation, and 55,356 (14%) died.

**Table 1:** Patient demographics and covariates.

	All individuals	Diabetic	Nondiabetic	*p* Value
Total individuals	402,388	66,082	336,306	
Male	214,161 (53%)	35,486 (54%)	178,675 (53%)	*p* < 0.01
Female	188,227 (47%)	30,596 (46%)	157,631 (47%)	*p* < 0.01
Age	46.16 ± 15.55	57.74 (1305%)	43.89 ± 14.98	*p* < 0.01
Pregnant	2838 (1%)	106 (0%)	2732 (1%)	*p* < 0.01
Native	385,671 (96%)	63,284 (96%)	322,387 (96%)	*p* = 0.32
Hypertension	81,604 (20%)	35,450 (54%)	46,154 (14%)	*p* < 0.01
Obesity	77,003 (19%)	18,454 (28%)	58,549 (17%)	*p* < 0.01
Smoking	29,996 (7%)	5276 (8%)	24,720 (7%)	*p* < 0.01
Pneumonia	88,064 (22%)	28,105 (43%)	59,959 (18%)	*p* < 0.01
ICU	10,285 (3%)	3509 (5%)	6776 (2%)	*p* < 0.01
Intubation	23,670 (6%)	8433 (13%)	15,237 (5%)	*p* < 0.01
Death	55,356 (14%)	21,129 (32%)	34,227 (10%)	*p* < 0.01

Abbreviation: ICU, intensive care unit.

Table 2 describes the univariable odds of pneumonia, ICU stay, intubation, and death in hospitalized adult COVID‐19 patients with comorbid diabetes. There was a 240% increased chance of getting pneumonia (odds ratio [OR]: 3.4, confidence intervals [CI]: 3.35−3.47, *p* < 0.01), 9% increase of ICU requirements (OR: 1.09, CI: 1.05−1.14, *p* < 0.01), 21% increase in intubation rates (OR: 1.21, CI: 1.17−1.24, *p* < 0.01), and a 315% increased chance of dying (OR: 4.15, CI: 4.07−4.23, *p* < 0.001) if the patient had comorbid diabetes compared to those that did not.

**Table 2:** Univariable model for outcomes of COVID‐19 on diabetes.

	Odds ratio	95% confidence interval	*p* Value
Pneumonia	3.4	(3.35−3.47)	*p* < 0.01
ICU	1.09	(1.05−1.14)	*p* < 0.01
Intubation	1.21	(1.17−1.24)	*p* < 0.01
Death	4.15	(4.07−4.23)	*p* < 0.01

Abbreviation: ICU, intensive care unit.

In Table 3, it can be seen that even after controlling for possible confounders such as age, sex, and comorbidities, patients with diabetes had 80% (OR: 1.80, CI: 1.76−1.84, *p* < 0.01) increased risk for pneumonia.

**Table 3:** Multivariable regression for pneumonia on diabetes in COVID‐19 patients.

	Odds ratio	95% CI	*p* Value
Age	1.05	(1.04−1.06)	*p* < 0.01
Sex	1.59	(1.56−1.62)	*p* < 0.01
Native	1.34	(1.26−1.43)	*p* < 0.01
Diabetes	1.8	(1.76−1.84)	*p* = 0.65
Hypertension	1.19	(1.17−1.22)	*p* < 0.01
Obesity	1.46	(1.43−1.49)	*p* < 0.01
Smoking	1.04	(1.01−1.07)	*p* = 0.02

In Table 4, even after controlling for possible confounders such as age, sex, and comorbidities, patients with diabetes had 9% increased risk for ICU requirement (OR: 1.09, CI: 1.04−1.14, *p* < 0.01).

**Table 4:** Multivariable regression for ICU on diabetes in COVID‐19 patients.

	Odds ratio	95% CI	*p* Value
Age	1	(1−1)	*p* < 0.01
Sex	1.25	(1.2−1.31)	*p* < 0.01
Native	1.07	(0.92−1.25)	*p* = 0.36
Diabetes	1.09	(1.04−1.14)	*p* < 0.01
Hypertension	0.96	(0.92−1.01)	*p* = 0.13
Obesity	1.39	(1.33−1.46)	*p* < 0.01
Smoking	0.93	(0.86−1)	*p* = 0.05

Abbreviation: ICU, intensive care unit.

In Table 5, even after controlling for possible confounders such as age, sex, and comorbidities, patients with diabetes had 7% increased risk for intubation (OR: 1.07, CI 1.04−1.11, *p* < 0.01).

**Table 5:** Multivariable regression for intubation on diabetes in COVID‐19 patients.

	Odds ratio	95% CI	*p* Value
Age	1.02	(1.02−1.02)	*p* < 0.01
Male	1.27	(1.23−1.31)	*p* < 0.01
Native	0.7	(0.61−0.78)	*p* < 0.01
Diabetes	1.07	(1.04−1.11)	*p* < 0.01
Hypertension	1.11	(1.08−1.15)	*p* < 0.01
Obesity	1.2	(1.16−1.24)	*p* < 0.01
Smoking	1.04	(0.99−1.10)	*p* = 0.15

In Table 6, even after controlling for possible confounders such as age, sex, and comorbidities, patients with diabetes had 88% increased risk for death (OR: 1.88, CI: 1.84−1.93, *p* < 0.01) compared to those without diabetes.

**Table 6:** Multivariable regression for death on diabetes in COVID‐19 patients.

	Odds ratio	95% CI	*p* Value
Age	1.08	(1.08−1.08)	*p* < 0.01
Male	1.87	(1.83−1.91)	*p* < 0.01
Native	1.23	(1.13−1.32)	*p* < 0.01
Diabetes	1.88	(1.84−1.93)	*p* < 0.01
Hypertension	1.32	(1.30−1.36)	*p* < 0.01
Obesity	1.5	(1.46−1.53)	*p* < 0.01
Smoking	0.99	(0.95−1.03)	*p* = 0.518

## 4 DISCUSSION

We studied the population of Mexico in a sizable, retrospective, cross‐sectional, multistage cohort research. According to the study's findings, persons with COVID‐19 who tested positive for the SARS‐CoV‐2 antigen and had concomitant diabetes had an 88% higher chance of dying and an 80% higher probability of pneumonia, 9% higher chance of ICU use, and 7% higher chance of intubation. According to the study, individuals with COVID‐19 had a high prevalence of concomitant conditions, such as hypertension (20%), diabetes (30%), and obesity (19%). These results are in line with earlier studies on US adults that found people with diabetes are more likely to experience severe sickness and COVID‐19‐related problems.^31^, ^32^, ^33^

In our study, the prevalence rates of diabetes among COVID‐19 patients are comparable to those reported in other studies, such as 21.6% among Wuhan's hospitalized COVID‐19 patients.^15^ In the wake of the COVID‐19 pandemic, Mexico has seen an increase in the number of diabetes cases.^34^, ^35^, ^36^ Diabetes has been linked to worse outcomes in COVID‐19 patients in other studies.^37^, ^38^, ^39^ This includes not only a higher risk of severe illness, but also complications from COVID‐19.^40^ Kumar et al. conducted a meta‐analysis where out of 33 PubMed studies on COVID‐19 and diabetes, 90% of COVID‐19 patients with diabetes had an increased risk of death (OR: 1.90, CI: 1.37−2.64, *p* < 0.01).^41^ Al‐Salameh et al.‘s cohort study will in 2020 included 433 patients examined with multivariate analysis, which revealed that diabetics with COVID‐19 had a higher risk of death, ICU admission, and prolonged hospital stays.^42^ A study in Western Sydney showed that patients with diabetes had 6% increase in mortality, 8% increase in ICU requirement, and 6.6 day increase in length of stay (*p* < 0.01).^43^

Diabetes has also been linked to an increased risk of upper respiratory infections (URIs) like the common cold and influenza in addition to COVID‐19.^44^, ^45^, ^46^, ^47^ This increased risk may be caused by a number of biological and pathological mechanisms. Inflammation and damage to blood vessels and tissues, including those in the respiratory system, can result from high blood sugar levels, making it more difficult for the body to fight off infections.^48^ The body's ability to mount an immune response against viral infections can be hindered by these comorbidities, which can disrupt the normal function of the immune system.^49^, ^50^, ^51^ The increased risk of pneumonia, ICU stay, intubation, and death among diabetic patients in the Mexican cohort may be explained by the weakened immune system and association with other comorbidities.^52^, ^53^, ^54^

The association between diabetes and worse outcomes in URIs has been attributed to a number of epidemiological explanations, one of which is COVID‐19.^55^, ^56^ Other comorbidities, such as hypertension and obesity, were found to be associated with the diabetes cohort in this study (*p* < 0.01). However, despite these confounding factors being taken into account, diabetes continued to have a significant impact on COVID‐19 outcomes. Numerous studies have demonstrated that COVID‐19 patients who also have conditions like hypertension, diabetes, and obesity typically experience worse outcomes.^55^, ^57^, ^58^ The body's capacity to mount an immune response against viral infections can be hindered by these comorbidities, which can disrupt the immune system's normal function.^59^, ^60^

## 5 STRENGTHS

Our research has a number of advantages. The large sample size, which increased statistical power and made it possible to identify significant differences between groups, is a major strength. We also used a Ministry of Health‐validated sample, which made our results more reliable. We were able to assess the strength of the relationship between diabetes and COVID‐19 outcomes thanks to our method of analysis, which correctly took into account confounding factors. Additionally, the study was representative of the adult population in Mexico thanks to the use of 475 monitoring units throughout the country.

## 6 LIMITATIONS

When interpreting the findings, it is important to take into account a number of this study's limitations. It cannot demonstrate causation because it is a retrospective and observational study. Additionally, the study did not take into account temporal aspects of diabetes, making it challenging to comprehend how COVID‐19 affected the patient's diabetes status over time. Lastly, the data were gathered from the Mexican population, so the large sample size may not be representative of the diabetes and COVID‐19 patients worldwide. As a result, it may not be possible to apply the findings to other populations.

## 7 CONCLUSION

All outcomes—pneumonia, ICU admission, intubation, and death—were higher in patients with diabetes and COVID‐19 who were hospitalized. To learn more about how diabetes affects COVID‐19 outcomes and to develop practical ways to reduce the risk of severe illness and complications in the Mexican population, additional prospective studies are required.

## AUTHOR CONTRIBUTIONS

**Alexander A. Huang**: Conceptualization; formal analysis; funding acquisition; investigation; methodology; resources; software; supervision; writing—original draft; writing—review and editing. **Samuel Y. Huang**: Conceptualization; data curation; formal analysis; investigation; methodology; project administration; software; supervision; visualization; writing—original draft; writing—review and editing.

## CONFLICT OF INTEREST STATEMENT

The authors declare no conflict of interest.

## ETHICS STATEMENT

The methods behind acquisition and analysis of the data are described by the Epidemiological Surveillance System for Viral Respiratory Diseases of the Mexican Ministry of Health. The authors consent to participate in peer review and consent to publish.

## TRANSPARENCY STATEMENT

The lead author Samuel Y. Huang affirms that this manuscript is an honest, accurate, and transparent account of the study being reported; that no important aspects of the study have been omitted; and that any discrepancies from the study as planned (and, if relevant, registered) have been explained.

## Data Availability

The data from this cohort can be found on COVID‐19 database is publicly and freely available without restriction on the Mexican Federal Government website.
